# Four-Dimensional Computed Tomographic Image Evaluation of the Change of Condylar Movement With and Without Implant-Supported Removable Prostheses in the Fibular Mandible: A Pilot Case Series Report

**DOI:** 10.7759/cureus.39419

**Published:** 2023-05-23

**Authors:** Junya Yamashita, Kazunobu Hashikawa, Yasumasa Kakei, Toshinori Sekitani, Masaya Akashi

**Affiliations:** 1 Oral and Maxillofacial Surgery, Kobe University Graduate School of Medicine, Kobe, JPN; 2 Plastic Surgery, Nagoya University Graduate School of Medicine, Nagoya, JPN; 3 Radiological Technologist, Osaka College of High Technology, Kobe, JPN

**Keywords:** free fibula flap reconstruction, segmental mandibulectomy, condylar protrusion, four-dimensional computed tomography (4dct), implant-supported removable prostheses (isrp)

## Abstract

Implant-supported removable prostheses (ISrP) improve the quality of life, especially in patients who underwent mandibular reconstruction, but few studies have focused on the effect of ISrP in the fibular mandible on the function of the temporomandibular joint. The purpose of this pilot case series was to determine the usefulness of four-dimensional computed tomography (4DCT) images for the evaluation of differences in condylar movements with and without ISrP. Three patients who underwent ISrP following segmental mandibulectomy and free-flap reconstruction were evaluated. The participants were instructed to masticate a cookie during the 4DCT scan. The distance between the most anterior and posterior positions of the condyles on the sagittal view of the 4DCT images during the chewing of the cookies was measured and compared with and without ISrP. 4DCT revealed changes in the distances of condylar protrusion with and without wearing ISrP, but there were no obvious differences among the three patients. The 4DCT motion analysis was useful for the evaluation of the effect of wearing ISrP on condylar movements during mastication in patients with mandibular reconstruction and may become a useful objective evaluation method for the functional evaluation of ISrP.

## Introduction

It is believed that dental rehabilitation with osseointegrated implants in patients who underwent jaw bone resection and reconstruction improves oral function, oral diet achievement, and oral health-related quality of life [1, 2]. Implant-supported removable prostheses (ISrP) are considered the best treatment option for dental rehabilitation in patients who underwent mandibular reconstruction [1]. However, there are no standard measurements to evaluate the functional outcome of ISrP [3]. Previous studies have used self-assessments by patients or subjective evaluations by surgeons [1, 2, 4]. Although there are objective evaluation methods for ISrP, such as a standardized masticatory performance test using peanuts and the occlusal force test [3], the development of an objective evaluation method for the effect of ISrP on function is necessary.

Recently, it was reported that four-dimensional computed tomography (4DCT) can visualize the changes in mandibular movement in reconstructed mandibles [5, 6]. The purpose of the current pilot case series report was to determine whether 4DCT images are useful for the evaluation of differences in condylar movements with and without the use of ISrP.

## Case presentation

Three patients who underwent segmental mandibulectomy and simultaneous reconstruction with a free fibular osteocutaneous flap at Kobe University Hospital were enrolled in this study. Additionally, 4DCT examinations were performed as routine postoperative follow-up imaging to monitor tumor recurrence or osteoradionecrosis. The 4DCT examination was performed with an Aquilion ONE (Canon Medical Systems Corporation, Otawara, Japan) at the Kobe University Hospital (tube current, 10 mA; tube voltage, 120 kV; scanning time 5.25 s; rotation time: 0.35 s/rotation; slice thickness: 0.5 mm; field of view, 220 mm). All images were acquired axially with a 320-detector row CT scanner to allow for multiple phases of unenhanced 3D volume acquisition with 16 cm coverage. The patient's forehead was fixed with tape to prevent bodily movement. They were instructed to chew a cookie during the scan. CT scans were performed with and without ISrP. The gantry was angled to limit radiation to the eyes, and the inferior aspect of the field of view was tailored to minimize radiation to the thyroid. The exposure doses in all study participants were within the notification values recommended by the American Association of Physicists in Medicine (AAPM) Working Group on Standardization of CT Nomenclature and Protocols for 4DCT. For image post-processing, a volume rendering (VR) image was generated using a commercial software (Ziostation2; AMIN Inc., Tokyo, Japan).

The maximum distance between the most anterior and posterior positions of the posterior portion of the condyle during mastication was measured using sagittal multiplanar reconstruction (MPR) images. The number of strokes during the scanning was counted. The percentage of the larger protrusion (i.e., the percentage of strokes with protrusive distances exceeding the mean values) was also calculated. All measurements were performed by one oral and maxillofacial surgeon (author J.Y.) in a blinded manner. The patient provided written informed consent to participate in this study after receiving a full explanation of the purpose and structure of the study, which had already been approved by the Medical Ethics Committee of Kobe University (No. B200052). This article was previously posted to the medRxiv preprint server on April 27, 2020.

**Figure 1:**
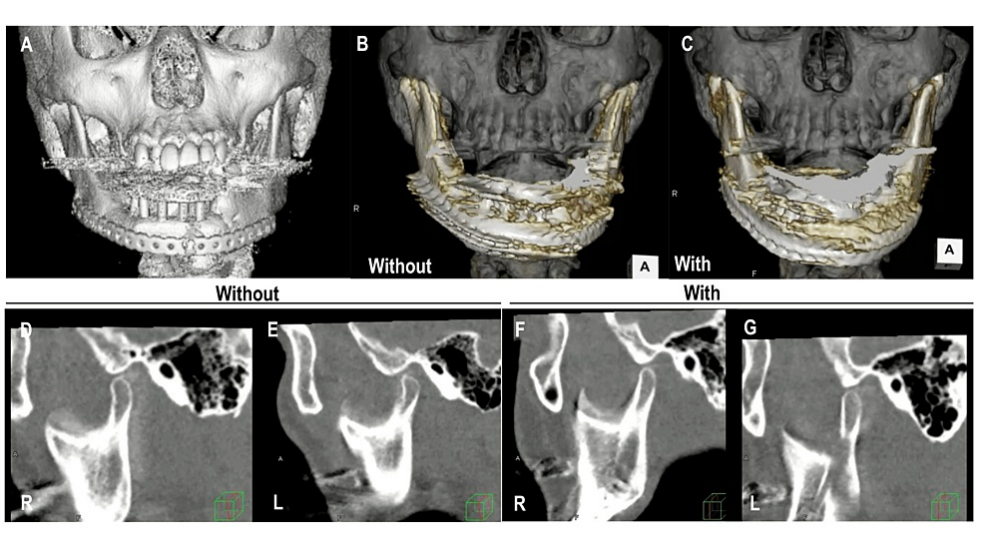
4DCT images (A) Frontal three-dimensional computed tomography (CT) image of patient two; frontal volume rendering images without (B) and with (C) implant-supported removable prostheses. Still images of sagittal four-dimensional computed tomography (D-G). 4DCT - four-dimensional computed tomography

Participants' characteristics are presented in Table 1. Representative images of patient two are shown in Figure 1. Figures 1B and C show coronal VR images with and without ISrP. Figures 1D-G show sagittal MPR images of condylar protrusive movements with and without ISrP.

**Table 1:** Comparison of condylar protrusion distance with and without implant-supported removable prostheses in the fibular mandible RT - radiotherapy; CDDP - cisplatin * Time interval between the fibula flap deconstruction and four-dimensional computed tomographic evaluation.

Case	Gender	Age	Pathological diagnosis	RT/ dose (Gy)	Chemotherapy	Extent of mandibulectomy	Time interval (months)^*^
1	M	52	Gingival squamous cell carcinoma	-	-	Right ramus-right premolar	77
2	M	61	Odontogenic carcinoma	-	-	Right angle-left premolar	61
3	M	67	Osteoradionecrosis	Conventional/66	CDDP	Right angle-right premolar	49

**Figure 2:**
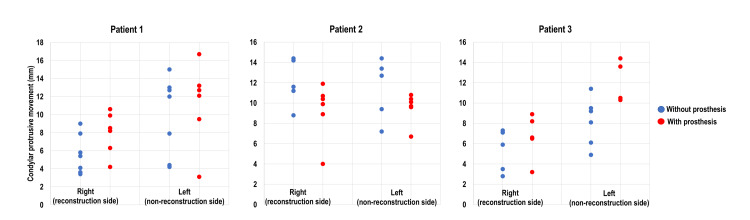
Changes in the distances of condylar protrusive movement with and without implant-supported removable prostheses

Figure 2 shows the changes in the condylar protrusion distances in the three patients. In patients one and three, the distances of condylar protrusion with ISrP were larger than those without ISrP, especially on the non-reconstructed sides. In contrast, the maximum amount of condylar protrusion with ISrP was smaller than that without ISrP in patient two. The median values of the distance of condylar protrusion were as follows: patient one right 5.4 mm and left 12 mm without ISrP, right 8.4 mm and left 12.7 mm with ISrP; patient two right 11.6 mm and left 12.7 mm without ISrP, right 9.9 mm and left 9.7 mm with ISrP; patient three right 6.5 mm and left 8.7 mm without ISrP, right 6.6 mm and left 13.6 mm with ISrP. The mean values of the distance of the condylar protrusion were as follows: patient one right 5.6 mm and left 9.9 mm without ISrP, right 8 mm and left 11.5 mm with ISrP; patient two right 12 mm and left 11.4 mm without ISrP, right 9.2 mm and left 9.6 mm with ISrP; patient three right 5.6 mm and left 8.2 mm without ISrP, right 6.7 mm and left 12.5 mm with ISrP.

The number of strokes during scanning and the percentage of larger protrusive distances above the mean values are listed in Table 2. Few changes in strokes during scanning were found in three patients. The percentage of larger protrusions of condyles increased with ISrP, except for the right (reconstructed) side in patient three.

**Table 2:** Comparison of condylar protrusion distance with and without implant-supported removable prostheses in the fibular mandible ISrP - implant-supported removal prosthesis * The percentage of strokes with protrusive distances exceeded the mean values.

Case	Number of strokes during scanning				Percentage of larger protrusion of condyle (%)*			
	Without ISrP		With ISrP		Without ISrP		With ISrP	
	Right	Left	Right	Left	Right	Left	Right	Left
1	7	7	6	7	43	57	67	71
2	5	5	7	7	40	60	57	71
3	6	6	5	5	67	50	40	60

No remarkable changes in condylar protrusion with or without ISrP were found among the three patients. Patient one had a history of segmental mandibulectomy, neck dissection, and fibula flap reconstruction for gingival cancer and did not undergo radiation therapy. Patient three had a history of concomitant chemoradiotherapy for oropharyngeal cancer and subsequent segmental mandibulectomy and fibula flap reconstruction for mandibular osteoradionecrosis. Although the surgical invasion of patient three was the lowest among the three patients, it was affected by irradiation. We found a similar condylar protrusion of the reconstructed side with and without ISrP in patients one and three, whereas the ranges of condylar protrusion of the non-reconstructed side were smaller in patients with ISrP than in those without ISrP in patients two and three. The number of missing teeth and the extent of the segments were similar in patients one and three, but larger in patient two than in patients one and three. Condylar movements during mastication are probably affected by the area and the number of missing teeth, irradiation, and the extent of defects of the mandible and the surrounding soft tissue, such as the masticatory muscle.

## Discussion

ISrP in the reconstructed mandibles contributes to the improvement of patients' quality of life, whereas there is no strong evidence to support the effect of ISrP because of the lack of objective evaluation methods of function [4]. This pilot case series report reveals a novel finding that wearing ISrP influences condylar movements during mastication, indicating the potential usefulness of 4DCT as a functional evaluation method for ISrP in patients who undergo mandibular reconstruction.

The influence of the loss of posterior teeth on the condyle has been a controversial issue. It was previously reported that prosthetic rehabilitation significantly changed the condylar position in women with good general health [7]. In this case series, changes in the distances of condylar protrusion with and without ISrP were observed in three patients. However, no obvious trend was detected. This may be due to not only the small number of patients but also the heterogeneity of patients, such as the resection extent of masticatory muscle and nerves, irradiation, and the number of residual teeth.

There are some important studies that have evaluated the effects of ISrP. Roumanas et al. [8] analyzed the function with standardized masticatory performance in patients who underwent segmental mandibulectomy with simultaneous free fibular flap reconstruction and reported that the impairment in masticatory function due to surgery was evident before and after surgery. Conventional removable prostheses significantly improve masticatory performance on the reconstruction sides [8]. ISrP greatly increased the number of patients who masticated the test food on the reconstruction side compared with the conventional removable prosthesis [8]. Notably, the mean values of masticatory performance on the reconstruction side with ISrP were significantly greater than those with the conventional removable prosthesis [8]. Interestingly, Roumanas et al. [8] indicated that good masticatory performance with fewer strokes during chewing until swallowing might be due to ISrP wearing [8]. In contrast, the number of strokes with and without ISrP did not change in this pilot study. Fueki et al. [9] reported that the electromyographic activity of the masseter muscles on the reconstructed side increased significantly from the conventional removable prosthesis to ISrP. A previous study reported that the side with the smaller condylar protrusion tends to be the habitual masticatory side [10]. This pilot report could not identify the characteristic tendency of changes in the percentage of larger protrusions.

Finally, there were some limitations in the current pilot report. First, this report included only three patients whose primary diseases, treatment history, and extent of mandibular defects were heterogeneous because the number of patients who underwent mandibulectomy and osseous reconstruction and subsequently completed ISrP was very limited [11]. Second, the current pilot report did not compare condylar movement during cookie chewing between ISrP and a conventional removal prosthesis. Therefore, the effect of dental implant support on prosthesis stability and mastication could not be determined. Third, the habitual masticatory side was not confirmed in any patient. Finally, additional radiation exposure and artifacts interfering with the motion analysis were observed. However, 4DCT motion analysis provides important clinical information that cannot be detected by any other modality. Refinement of 4DCT analysis may become an objective evaluation method for mandibular reconstruction and dental rehabilitation.

## Conclusions

Although 4DCT could visualize changes in the distances of condylar protrusion with and without wearing ISrP, there were no obvious differences among the three patients. Further investigations are necessary to clarify the usefulness of the 4DCT motion analysis for the evaluation of condylar movements during mastication in patients wearing ISrP.

## References

[1] Attia S, Wiltfang J, Streckbein P (2019). Functional and aesthetic treatment outcomes after immediate jaw reconstruction using a fibula flap and dental implants. J Craniomaxillofac Surg.

[2] Nguyen TT, Eo MY, Myoung H, Kim MJ, Kim SM (2020). Implant-supported fixed and removable prostheses in the fibular mandible. Int J Implant Dent.

[3] Wijbenga JG, Schepers RH, Werker PM, Witjes MJ, Dijkstra PU (2016). A systematic review of functional outcome and quality of life following reconstruction of maxillofacial defects using vascularized free fibula flaps and dental rehabilitation reveals poor data quality. J Plast Reconstr Aesthet Surg.

[4] Raoul G, Ruhin B, Briki S (2009). Microsurgical reconstruction of the jaw with fibular grafts and implants. J Craniofac Surg.

[5] Akashi M, Shibuya Y, Takahashi S (2016). Four-dimensional computed tomography evaluation of jaw movement following mandibular reconstruction: a pilot study. J Craniomaxillofac Surg.

[6] Akashi M, Sekitani T, Ohtsuki Y (2017). Axial four-dimensional computed tomographic images to analyze crosswise differences in protrusive condylar movement in patients who underwent mandibulectomy and free flap reconstruction. J Craniomaxillofac Surg.

[7] Amorim VC, Laganá DC, de Paula Eduardo JV, Zanetti AL. (2003). Analysis of the condyle/fossa relationship before and after prosthetic rehabilitation with maxillary complete denture and mandibular removable partial denture. J Prosthet Dent.

[8] Roumanas ED, Garrett N, Blackwell KE (2006). Masticatory and swallowing threshold performances with conventional and implant-supported prostheses after mandibular fibula free-flap reconstruction. J Prosthet Dent.

[9] Fueki K, Roumanas ED, Blackwell KE (2014). Effect of implant support for prostheses on electromyographic activity of masseter muscle and jaw movement in patients after mandibular fibula free flap reconstruction. Int J Oral Maxillofac Implants.

[10] Hiyama S, Ono T, Ishiwata Y, Kato Y, Kuroda TJ. (2000). The relationship between temporomandibular joint mobility and habitual chewing side or symptomatic side of temporomandibular disorders. Jpn Soc TMJ.

[11] Kwakman JM, Freihofer HP, van Waas MA (1997). Osseointegrated oral implants in head and neck cancer patients. Laryngoscope.

